# Surface Modification of Bioresorbable Phosphate Glasses for Controlled Protein Adsorption

**DOI:** 10.1021/acsbiomaterials.1c00735

**Published:** 2021-08-12

**Authors:** Ngoc Bao Hyunh, Cristina Santos Dias Palma, Rolle Rahikainen, Ayush Mishra, Latifeh Azizi, Enrica Verne, Sara Ferraris, Vesa Pekka Hytönen, Andre Sanches Ribeiro, Jonathan Massera

**Affiliations:** † Laboratory of Biomaterials and Tissue Engineering, Faculty of Medicine and Health Technology, Tampere University, Korkeakoulunkatu 3, 33720 Tampere, Finland; ‡ Laboratory of Biosystem Dynamics, Faculty of Medicine and Health Technology, Tampere University, 33520 Tampere, Finland; § Laboratory of Protein Dynamics, Faculty of Medicine and Health Technology, Tampere University, Arvo Ylpön katu 34, 33520 Tampere, Finland; ∥ Laboratory of Biomaterials, Department of Applied Science and Technology, Politecnico di Torino, 24 Corso Duca Degli Abruzzi, 10129 Torino, Italy; ⊥ Fimlab Laboratories, Biokatu 4, 33520 Tampere, Finland

**Keywords:** bioactive glass, phosphate, silicate, surface chemistry, protein adsorption

## Abstract

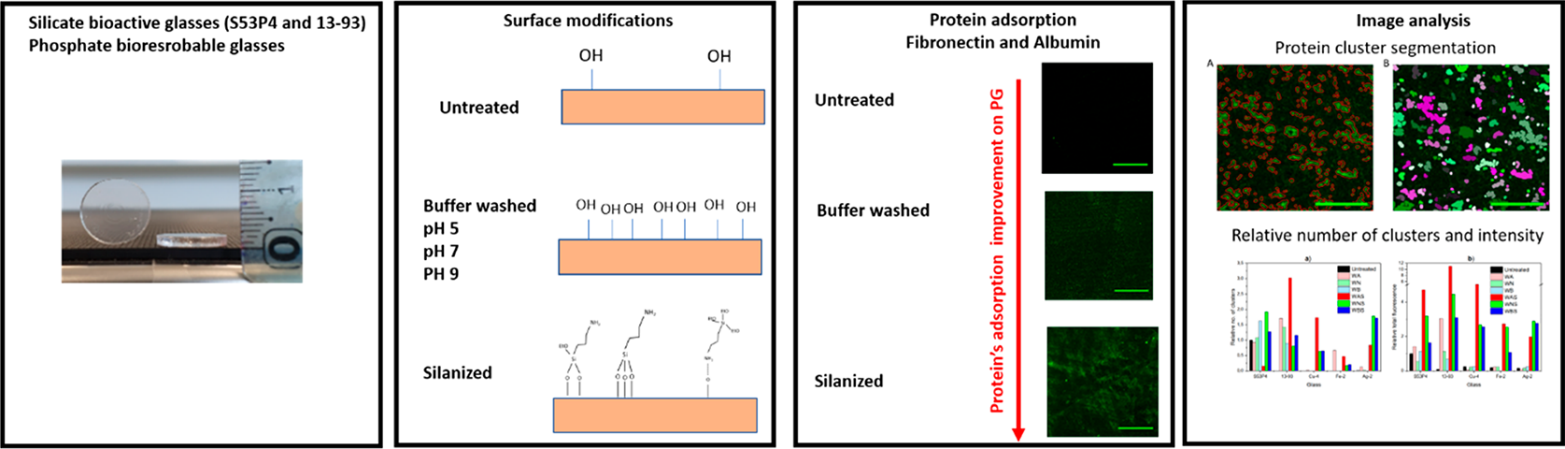

The traditional silicate bioactive glasses exhibit poor thermal processability, which inhibits fiber drawing or sintering into scaffolds. The composition of the silicate glasses has been modified to enable hot processing. However, the hot forming ability is generally at the expense of bioactivity. Metaphosphate glasses, on the other hand, possess excellent thermal processability, congruent dissolution, and a tailorable degradation rate. However, due to the layer-by-layer dissolution mechanism, cells do not attach to the material surface. Furthermore, the congruent dissolution leads to a low density of OH groups forming on the glass surface, limiting the adsorption of proteins. It is well regarded that the initial step of protein adsorption is critical as the cells interact with this protein layer, rather than the biomaterial itself. In this paper, we explore the possibility of improving protein adsorption on the surface of phosphate glasses through a variety of surface treatments, such as washing the glass surface in acidic (pH 5), neutral, and basic (pH 9) buffer solutions followed or not by a treatment with (3-aminopropyl)triethoxysilane (APTS). The impact of these surface treatments on the surface chemistry (contact angle, ζ-potential) and glass structure (FTIR) was assessed. In this manuscript, we demonstrate that understanding of the material surface chemistry enables to selectively improve the adsorption of albumin and fibronectin (used as model proteins). Furthermore, in this study, well-known silicate bioactive glasses (i.e., S53P4 and 13-93) were used as controls. While surface treatments clearly improved proteins adsorption on the surface of both silicate and phosphate glasses, it is of interest to note that protein adsorption on phosphate glasses was drastically improved to reach similar protein grafting ability to the silicate bioactive glasses. Overall, this study demonstrates that the limited cell/phosphate glass biological response can easily be overcome through deep understanding and control of the glass surface chemistry.

## 1 Introduction

The continually evolving field of tissue engineering aims at promoting tissue regeneration of damaged tissue using biomaterials in various forms, such as scaffolds, fibers, powders, etc. In the case of polymers and metals intended as biomaterials, biocompatibility is often poor, and they are expected to fulfill their function without causing adverse reactions in vivo.^1^ On the other hand, bioactive glasses have been shown to interact with the surrounding proteins and cells, invoking positive responses from the host tissue (osteoconduction and sometimes even osteostimulation).^2^ The field of bioactive glasses emerged after the discovery of Bioglass by Hench, attracting the attention of researchers toward bioactive glasses.^3^ Bioglass, also referred to as 45S5, is a silicate glass with composition 45.0SiO_2_–24.5CaO–24.5Na_2_O–6.0P_2_O_5_ (wt %), and it is still considered as a benchmark for bioactive glasses. This is attributed to its ability to form a strong bond with the bone.^3^ However, for bone tissue engineering, developing porous scaffolds to mimic the porous nature of bone is of paramount importance. Unfortunately, the high tendency of crystallization of bioactive glasses such as Bioglass and BonAlive inhibits their fabrication into porous scaffolds and fiber drawing during hot working processes.^4,5^ Additionally, the remnants of silicate bioactive glasses were found at the implantation site several years after the surgery.^6^ This is indeed undesirable, and the investigation of the long-term effects due to exposure to silica has been a matter of interest.^7^ Considering the above facts, the search for better biomaterials has continued, and among the alternatives, phosphate glasses (PGs) have emerged as a promising candidate for bioresorbable commercial devices.

PGs possess congruent dissolution, tailorable degradation rate,^8^ and good solubility toward metal ions, which may be used to impart unique properties to the glass.^9−12^ Additionally, PGs also have a wide thermal processing window, which enables them to be thermally processed into complex shapes and geometries.^13^ Therefore, phosphate glass fibers have been studied extensively for various applications.^13−16^

In previous study, the glass system *x*MO + (100 – *x*) (50P_2_O_5_ + 10Na_2_O + 20CaO + 20SrO) (mol %), where *x* and MO represent the dopant concentration and the dopant metal oxide, respectively, was studied in terms of its in vitro dissolution properties. Compared to the reference glass with *x* = 0 (referred to as Sr50), doping with metal oxides was found to change the degradation rate while maintaining a large thermal processing window for all of the glasses. The Ag- and Cu-doped glasses were also shown to possess antibacterial properties, in agreement with previous studies on such glasses.^10−17^

Furthermore, in cell culture experiments, metaphosphate glasses were found to support the growth of human gingival fibroblasts on their surface, though at a slower rate than the silicate glasses.^18^ While the cells attached, proliferated, and grew over a long time of culture, the early attachment of the cells, to the surface of the materials, was poor. This indicated that although the dissolution products of this glass were favorable for cell viability, the surface itself does not provide a substrate amenable to cell attachment.^18^ In addition to the above observations, Cu- and Ag-doped Sr50-based glasses have a higher dissolution rate than the Sr50 itself, and fast dissolving glasses have been shown to inhibit cell growth and proliferation.^7^ Therefore, there is a need to understand and tailor the surface characteristics of PG to improve cell attachment to their surface.

When a biomaterial is exposed to tissue or cell culture media, there is instantaneous adsorption of proteins on its surface. Protein deposition leads to the formation of a provisional matrix on the material. Eventually, when the cells approach the biomaterial, they interact with this provisional matrix of proteins at its surface, rather than the biomaterial itself. Thus, protein adsorption on the biomaterial’s surface is a critical step toward cell viability. Furthermore, in an effort to improve their bioactivity, surface functionalization of silicate glasses (SGs) has been widely studied in the past.^19^ Functional organosilanes such as (3-aminopropyl)triethoxysilane (APTS) are suitable choices for surface functionalization on biomaterial’s surface. For instance, APTS was functionalized on a glass substrate to immobilize DNA, allowing, in turn, DNA sequencing.^20^ In other studies, APTS was employed as a linker for the immobilization of bone morphogenetic protein 2 (BMP-2) and alkaline phosphatase enzyme.^21^ Detailed surface analysis of APTS-functionalized PG is reported in ref (22). In this study, the amount of APTS grafted on the surface of PG glasses, within the composition 50P_2_O_5_–(40–*x*)CaO–*x*SrO–10Na_2_O, with *x* varying from 0 to 40, was found to be constant, independently of the glass composition.^22^ The APTES grafting mechanism on silicate surface has also be reported in ref (23).

In view of the importance of protein adsorption on the biological compatibility and tissue integration of a biomaterial, we investigated the impact of surface treatment (change in surface charge and chemical structure) on the ability to adsorb model proteins, i.e., fibronectin and albumin. The surface charge was altered by washing the glass discs in acidic, neutral, or basic buffer solutions. Furthermore, APTS was deposited on the surface of these materials using the protocol described in ref (22), and its impact on protein adsorption was quantified with fluorescence microscopy and thorough image analysis. The SGs in this study are the well-known bioactive glasses S53P4 and 13-93. The PGs chosen are based on the glass system described earlier, having the general composition *x*MO + (100 – *x*) (50P_2_O_5_ + 10Na_2_O + 20CaO + 20SrO) (mol %), labeled Fe-2 (*x* = 2, MO = Fe_2_O_3_), Cu-4 (*x* = 4, MO = CuO), and Ag-2 (*x* = 2, MO = Ag_2_SO_4_).^10,17^

## 2 Materials and Methods

### 2.1 Preparation of the Glass Discs

SG S53P4 and 13-93 were prepared using analytical-grade SiO_2_ (Belgian quartz sand), MgCO_3_, CaCO_3_, K_2_CO_3_, Na_2_CO_3_ and (CaHPO_4_)·2H_2_O as raw materials (all raw materials purchased from Sigma-Aldrich, Saint Louis, MI). PGs within the composition *x*MO + (100 – *x*) (50P_2_O_5_ + 10Na_2_O + 20CaO + 20SrO) (mol %), where *x* and MO represent the dopant concentration and the dopant metal oxide, respectively, were prepared using Ca(PO_3_)_2_, Sr(PO_3_)_2_, NaPO_3_, and Fe_2_O_3_/CuO/Ag_2_SO_4_ as raw materials (Sigma-Aldrich, Saint Louis, MI). Ca(PO_3_)_2_ and Sr(PO_3_)_2_ used for making the batch were obtained beforehand by heating NH_4_H_2_PO_4_ with CaCO_3_ and SrCO_3_ in separate crucibles to 250 °C for 12 h, then to 650 °C for 12 h, and finally to 850 °C for 12 h at 1 °C/min to remove CO_2_, NH_3_, and H_2_O. Glass (45 g) was melted in a Pt crucible in air. The glass melting temperature was set based on previous melting protocols.^4,5,10,17^ The compositions of all of the studied glasses are presented in Table 1. After melting, the glass batch was cast into a preheated brass mold of diameter 12 mm and annealed for 15 h at (*T*_g_ – 15)°C to release the thermal stresses. After annealing, the rod was cut into 2 mm thick discs using a Low-Speed Diamond Wheel Saw, Model 650, South Bay Technology (San Clemente, CA). The glass discs were then polished to the same surface roughness with grit #320, #500, #800, #2400, and #4000 (Struers, Copenhagen, Denmark) before any surface treatments.

**Table 1:** Nominal Glass Compositions

	glass label	SiO_2_ (mol %)	CaO (mol %)	Na_2_O (mol %)	P_2_O_5_ (mol %)	SrO (mol %)	K_2_O (mol %)	MgO (mol %)	Ag_2_SO_4_ (mol %)	Fe_2_O_3_ (mol %)	CuO (mol %)
SG	S53P4	53.85	21.77	22.66	1.72						
	13-93	54.6	22.1	6.0	1.7		7.9	7.7			
PG	Ag-2		19.6	9.8	49	19.6			2		
	Fe-2		19.6	9.8	49	19.6				2	
	Cu-4		19.2	9.6	48	19.2					4

### 2.2 Preparation of the Buffer Solutions

The reagents used to prepare the buffer solutions were Tris base for the basic buffer (pH = 9.0), Tris base and Tris–HCl (Sigma-Aldrich, Saint Louis, MI) for the neutral buffer (pH = 7.4), and citric acid-sodium citrate for the acidic buffer solution (pH = 5.0). The solutions were filtered with 0.2 μm filter paper and autoclaved before use. All of the buffer solutions had an ionic concentration of 10 mM.

### 2.3 Washing and Silanization

The glasses were washed in acidic/neutral/basic buffer solution by immersing the glass disc in the buffer solution for 6 h at room temperature. The immersion time was selected based on previous experiments that showed that (1) for longer immersion time a thin, unstable, reactive layer could precipitate on phosphate glasses and (2) maximum changes in the ζ-potential and contact angle (compared to the bare glass) were recorded. The glass discs were then dried at room temperature in a laminar hood. Consequently, the silanization of the glass surface was carried out as per the protocol in ref (22) and are summarized below: The glass discs washed in acidic/neutral/basic buffer solution are referred to as WA/WN/WB and after silanization as WAS/WNS/WBS, respectively.

#### 2.3.1 Washing

Samples were immersed in acetone (95 vol %) for 5 min in a sonicator. Then, they were immersed 3 × 5 min in double-distilled water in a sonicator.

#### 2.3.2 Silanization

Washed glass discs were immersed in 150 mL of a solution containing 95 vol % ethanol and 35 μL of APTS (Sigma-Aldrich, Saint Louis, MI), leading to a concentration of 1 mmol/L, for 6 h, at RT.

#### 2.3.3 Drying/Rinsing

Samples were dried at 100 °C, for 1 h, to consolidate the bonding between the silane and glass surface. Samples were then rinsed three times with ethanol in a sonicator to remove any excess APTS sticking to the glass disc but not physically bonded. The samples were then dried again for 1 h at 100 °C.

### 2.4 Change in the Glass Network

The treated glass discs were analyzed by a PerkinElmer Spectrum One FTIR Spectrophotometer (PerkinElmer, Waltham, MA) in the attenuated total reflectance (ATR) mode. The IR spectra were recorded in the range of 600–1600 cm^–1^. The spectra were subsequently corrected for Fresnel losses and normalized to the band having the maximum intensity. All of the spectra were obtained as an average of eight scans with a resolution of 1 cm^–1^.

### 2.5 Contact Angle Measurements

The static contact angle was measured on both treated (only washed and washed+silanized) and untreated samples using a sessile droplet method on an Attension Theta contact angle meter (Biolin Scientific, Gothenburg, Sweden). A droplet of 3–4 μL of the buffer solution was set onto the surface of glass discs, and the image of the droplet was recorded with a high-speed camera. The contact angle of both sides of the droplet was obtained using software Attension Theta. A representative image of a drop on the surface of the S53P4-WBS is presented in Figure S1. The measurement was repeated thrice on different glass discs, and the values are presented here as mean ± standard deviation.

### 2.6 ζ-Potential

The ζ-Potential on the surface of the glass discs was measured with an electrokinetic analyzer (SurPASS, Anton Paar, Graz, Austria), which was equipped with an adjustable gap cell. Measurements were performed at physiological pH (7.4) in diluted simulated body fluid solution (SBF), prepared by dropwise addition of SBF to water, to reach pH 7.4 and conductivity around 15 mS/m.

### 2.7 Protein Grafting and Confocal Microscopy

Fluorescent-labeled proteins, bovine serum albumin (BSA) and human fibronectin, were grafted on the glass discs and kept in the dark for 24 h before imaging by confocal microscopy using a 488 nm laser and a 525/50 nm emission bandwidth.

Alexa Fluor 488 NHS Ester (Invitrogen, Thermo Fisher Scientific, MA) was used for labeling BSA (Sigma-Aldrich, Saint Louis, MI) and fibronectin (purified from human plasma using gelatin affinity chromatography) according to instructions of the manufacturer. The free dye was removed by extensive dialysis, and the amount of fluorophores per protein was quantified using UV/vis spectroscopy (1.04 and 8.07 dyes/protein for BSA and fibronectin, respectively).

The proteins were diluted in the acidic/neutral/basic buffer solutions to obtain a solution of concentration 10 μg/mL. It must be said that the pH range used here only goes from moderately acidic (5.0) to moderately basic (9.0). While the pH change may lead to protein denaturation, a limited impact is expected in the given pH range, as the pH is not a strong denaturing factor. Furthermore, based on the side chain p*K*_a_’s of the protein of investigation, no change in protonation is expected. Indeed, Marković et al. demonstrated by dichroism that fibronectin was not denaturated between pH 3 and 11.^24^ With regard to albumin, denaturation was reported at pH levels lower than 5 in ref (25) and was reported to be instantaneous at pH 4.^26^ At basic conditions, between pH 7 and 9, BSA and HSA are known to go through a subtle and gradual conformational change (N–B transition). Such transition is generally associated with the loss of molecular rigidity, which affects the N-terminal region and, thus, impacts ligand biding.^27^ Finally, it was shown in ref (28) that preconditioning bioactive glass (45S5) at various pH levels can strongly influence the adsorption of BSA. The glass substrates were washed with the buffer solutions prior to applying the protein solution. Then, a pair of 120 μm thick poly(dimethylsiloxane) (PDMS) strips were laid on the polystyrene (PS)-uncoated six-well plate to act as spacers. This was done to provide a suitable area (∼80–90 mm^2^) for the applied protein solution aliquot to reside under the glass disc and to form a uniform protein layer across the surface of the disc. The contact time was 30 min, and the volume of the drop was 20 μL. The glass discs were then washed thrice with 2 mL of phosphate-buffered saline (PBS) to remove any excess protein for 2 min using an orbital shaker at 250 rpm. Further, the discs were washed with distilled water, immediately mounted on glass slides with 10 μL of ProLong Diamond Antifade Mountant (Invitrogen, Thermo Fisher Scientific, MA), and cured in a dark place for 24 h at room temperature.

### 2.8 Quantitative Analysis of Fluorescent Images

All samples within each experiment were imaged using constant laser intensity and imaging parameters. Images were taken using z-stack (total thickness of a few micrometers) to ensure the best signals and partly compensate for the tilting surface of the glass, if applicable. From the images obtained by confocal microscopy, we extracted the number of clusters of fluorescent proteins as well as the total fluorescence in the image (sum of the intensity of all pixels) using ImageJ (Fiji). The segmentation of the clusters was done using software CellAging.^29^ To obtain the binary mask of the segmented clusters, we applied a two-dimensional (2D) adaptive threshold based on the local mean intensity of the neighborhood of each pixel.^30^ Next, the binary image is subjected to morphological operations to avoid oversegmentation, merging of clusters, and false-positive clusters. This was done using the functions “*bwareaopen*” (with an area opening of 20 pixels) and “*bwmorph*” (operations used were “*hbreak*,” “*open*,” and “*thicken*”) of the Image Processing Toolbox of MATLAB software, version R2016a.^31^ After automatic segmentation, when needed, the results were manually corrected, resulting in little to no errors. An example of the results from the segmentation of clusters is shown in Figure S2. Explanation regarding the error of measurement estimation can be found in Section S3.

The number of clusters and total fluorescence from the fluorescence images were obtained. When evaluating the impact of the surface treatments for each glass, the numerical values were normalized to the untreated S53P4 glass.

## 3 Results and Discussion

The aim of this study was to develop new surface treatment methods for phosphate glasses to improve the protein adsorption on their surface relative to commercially available silicate bioactive glasses S53P4 and 13-93. It is worth noting here that the glasses chosen for this study have dissimilar dissolution rates. 13-93 has been shown to dissolve much slower than S53P4 in vitro.^32^ Similarly, among the PGs, Fe-2 was found to degrade much slower in vitro than Ag-2 and Cu-4.^10,17^

Subsequently, the short-range structure, hydrophobicity, and ζ-potential were studied on the surface of the glasses, as a function of the stages of surface treatments. Furthermore, protein adsorption on the surface of the SG and PG in this study was determined by confocal microscopy to assess the effectiveness of the different surface treatments.

### 3.1 Structural Changes on the Surface due to Washing and Silanization

**Figure 1:**
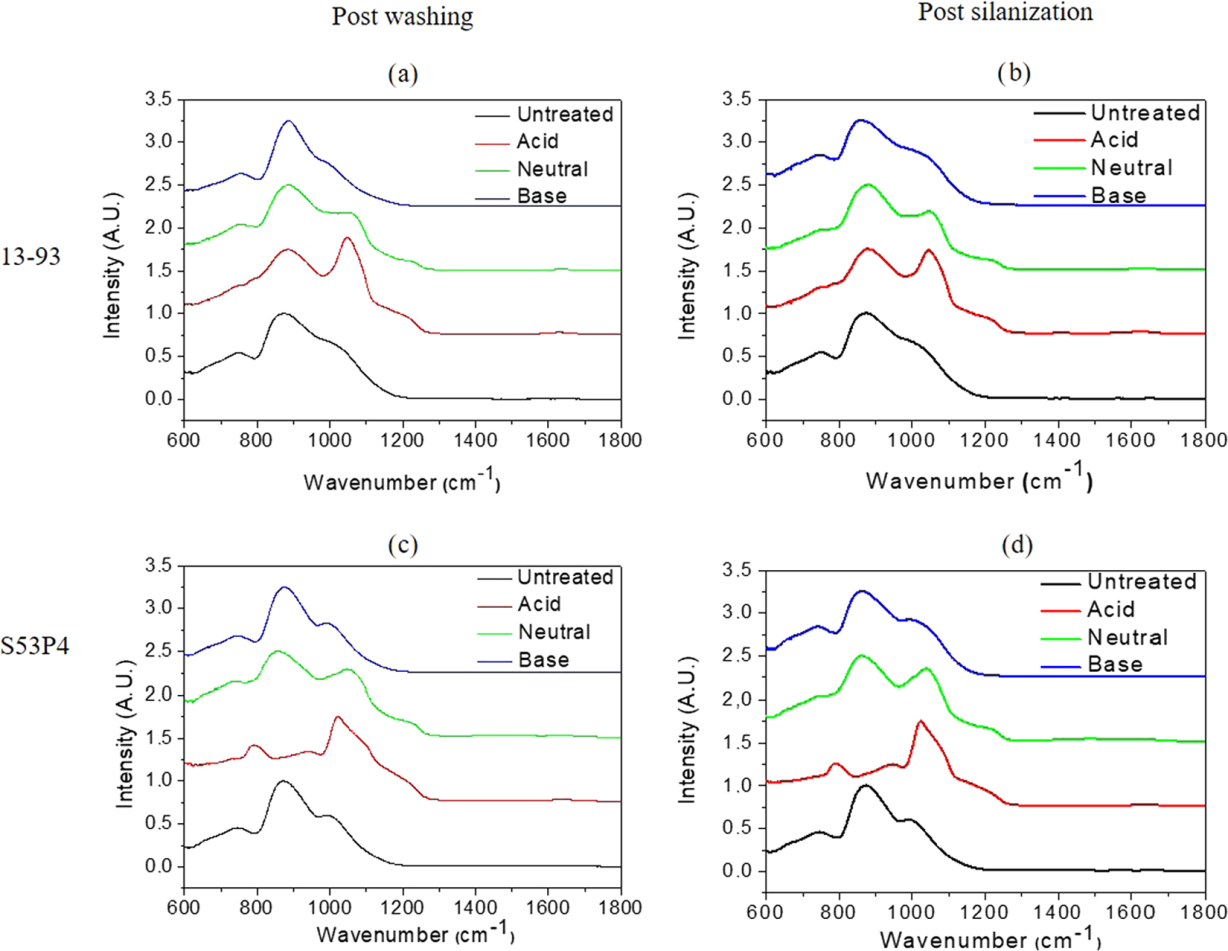
FTIR-ATR spectra of the silicate glasses 13-93 (a, b) and S53P4 (c, d), postwashing and postsilanization, in the three buffers.

**Figure 2:**
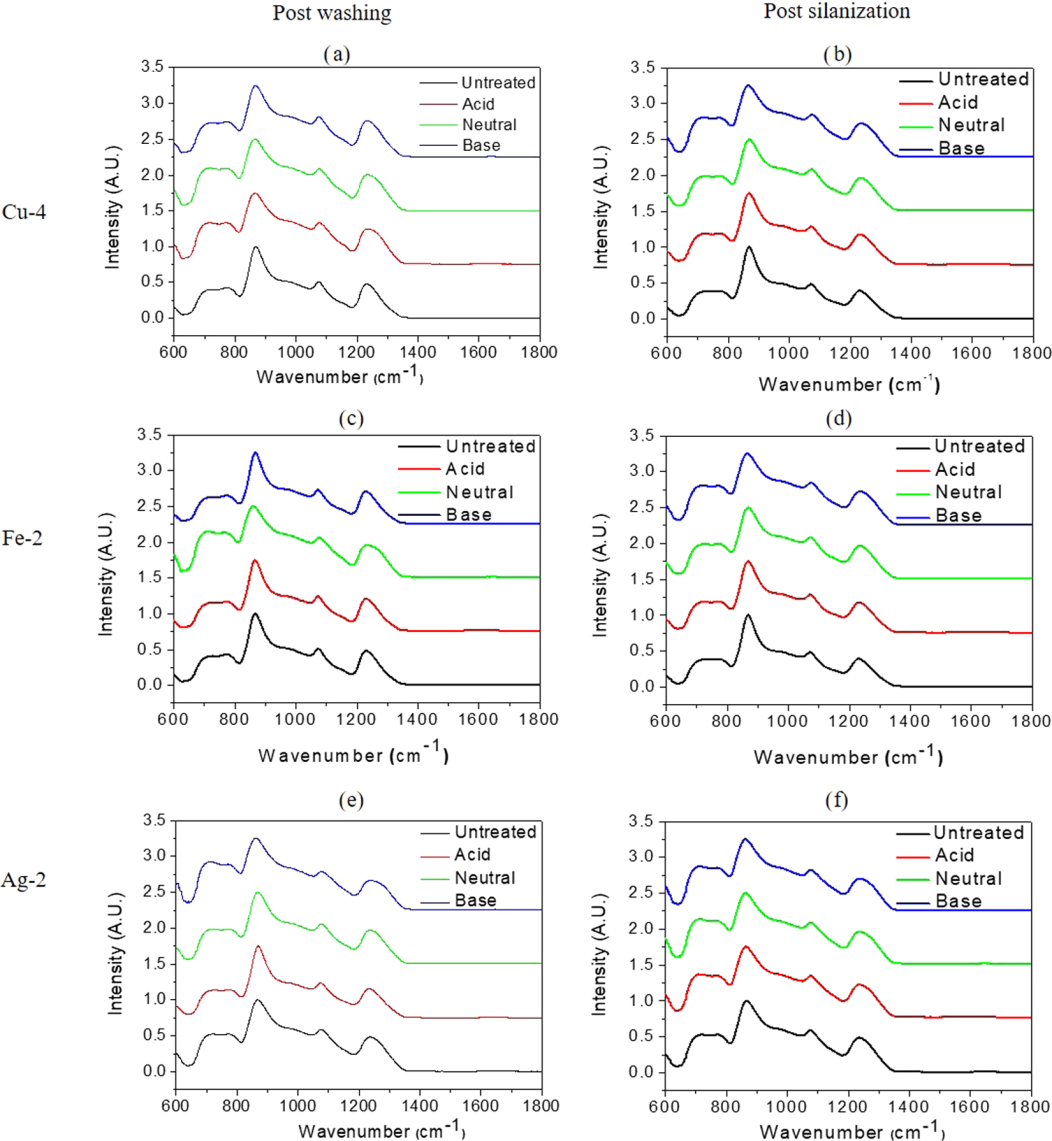
FTIR-ATR spectra of the phosphate glasses Cu-4 (a, b), Ag-2 (c, d), and Fe-4 (e, f), postwashing and postsilanization, in the three buffers.

Upon exposure to aqueous media of different pH levels, a change was expected in the short-range structure and surface charge on the glass surface. In Figures 1 and 2, the FTIR-ATR spectra of the SG and PG, respectively, are presented, postwashing (WA, WN, WB), and postsilanization (WAS, WNS, WBS). In Figure 1, the spectra of the untreated S53P4 and 13-93 depicted three absorption bands at 740, 870, and 992 cm^–1^.

These bands were assigned to Si–O–Si bending, Si–O–Si symmetrical stretching mode, and Si–O–NBO vibration, respectively.^33,34^ All of the spectra were normalized to the band at 870 cm^–1^. The spectra of the untreated glasses are typical of bioactive silicate glasses, where the structure is mainly composed of Q^2^ and Q^3^ units.^33−36^ The spectra of the WB and WN S53P4 and 13-93 samples remained largely unaffected. On the other hand, new bands appeared at 1045 and 1236 cm^–1^ in the spectra of WA samples for both S53P4 and 13-93 glasses. The new band at 1045 cm^–1^ is attributed to the P–O vibration mode from the formation of a calcium phosphate reactive layer assumed to be a hydroxyapatite (HA) layer.^37^ The shoulder at 1236 cm^–1^ was assigned to the Si–O–Si symmetrical stretching and indicated the creation of a silica-rich layer on the surface of the glass as reported earlier.^38,39^ Furthermore, a decrease in intensity of the band at 740 cm^–1^ and the rise of a new band at 790 cm^–1^, postwashing in neutral conditions, can be assigned to the C–O vibration mode in (CO_3_^2–^).^40^ The presence of carbonate vibration is characteristic of the formation of hydroxycarbonated apatite precipitation (HCA).^37^ Upon washing in acidic buffer solution, the change in the bands is further exacerbated. The bands at 740 and 870 cm^–1^ almost completely disappear at the expense of the bands at 790 and 1045 cm^–1^ (which further shifts to 1032 cm^–1^), indicating that, in an acidic buffer solution, the precipitation of HCA is faster. It is noteworthy that the change in the glasses surface structure is significantly faster for S53P4, as a consequence of its faster dissolution/reaction rate, when compared to that for glass 13-93. After silanization, no further changes in the spectra were evidenced for both glasses.

Figure 2 presents the FTIR-ATR spectra of the PG included in this study. The spectra of the based glasses, prior to any surface treatment, exhibit absorption bands at 708, 782, 865, 1078, and 1234 cm^–1^ and a shoulder at 980 cm^–1^. All of the spectra have been normalized to the band having a maximum intensity at 865 cm^–1^. All of the bands may be attributed to a classical metaphosphate glass structure.^41,42^ The band having the maximum intensity at 865 cm^–1^ can be assigned to the P–O–P asymmetric stretching in Q^2^ units.^43,44^ The bands at 708 and 782 cm^–1^ correspond to the P–O–P asymmetrical stretching modes,^45^ and the shoulder at 980 cm^–1^ and the band at 1078 cm^–1^ may be attributed to the symmetric and asymmetric vibrations of PO_3_^2–^ in Q^1^ units, respectively.^43,45−47^ Furthermore, the band at 1078 cm^–1^ is also attributable to the overlap between PO_3_ Q^1^ terminal groups and PO_2_ Q^2^ groups in the metaphosphate structure.^48^ Regardless of the pH of the buffer used for the washing step, or silanization, no significant change in the structural properties could be evidenced on the surface of the PG. This can be attributed to the congruent dissolution mechanism of PG, whereby the surface structure remains unaltered upon dissolution in aqueous media.

### 3.2 Change in Hydrophilicity due to Washing and Silanization

**Figure 3:**
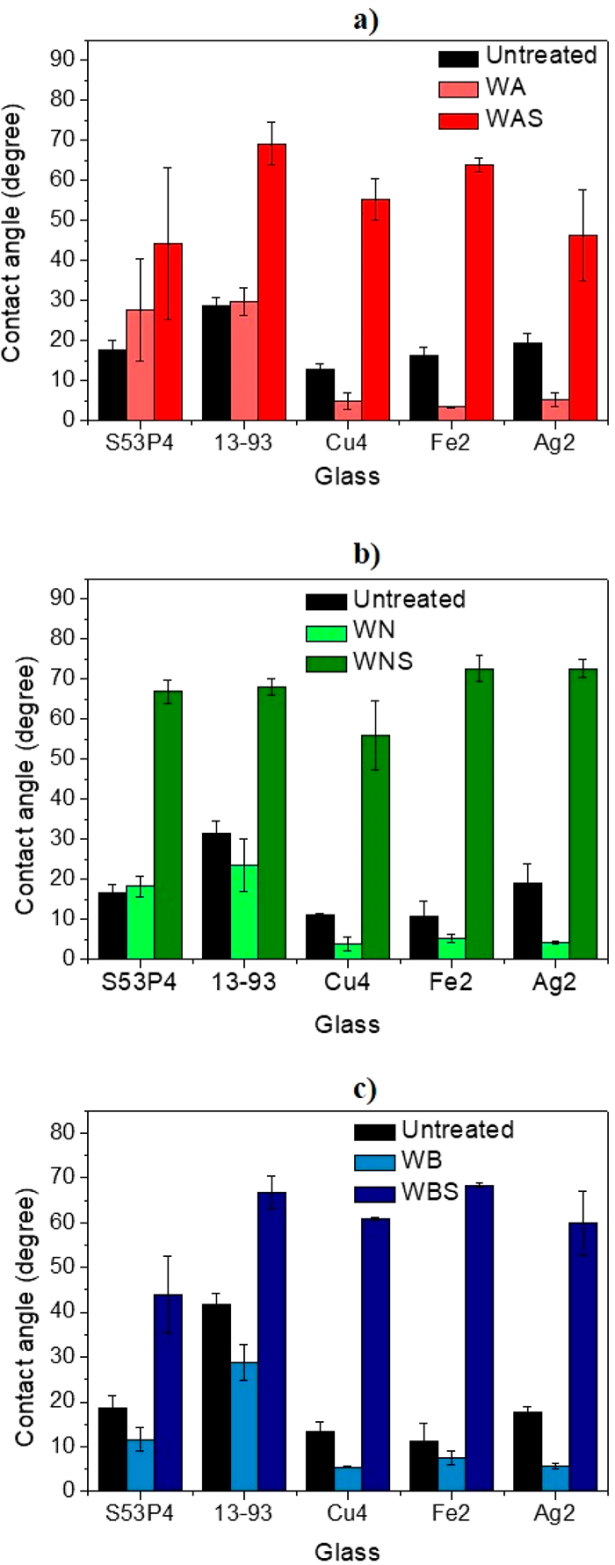
Contact angles of the silicate and phosphate glasses postwashing and postsilanization when the surface treatment is conducted with a buffer having (a) acidic, (b) neutral, and (c) basic pH.

Figure 3 shows the contact angle of a water droplet on the surface of the untreated, washed, and washed + silanized SG and PG.

As described in ref (22), the grafting of an APTS layer can be detected using contact angle measurements. Irrespective of the treatment, 13-93 has the highest contact angle among all of the glasses under investigation. In the untreated condition, all of the glasses presented a contact angle <20°. Exceptionally, the contact angle for the untreated 13-93 was ∼34 ± 7°, owing to the lower amount of −OH groups present on its surface as compared to that for S53P4.^32^ For both the SGs, the contact angle remained unchanged in the WA and WN conditions (Figure 3a,b, respectively). This is rather surprising as, in the case of SG, the contact angle was found to decrease upon washing with ethanol and distilled water.^19,21^ However, among the WB samples (Figure 3c), both the SGs presented a decrease in the contact angle, which was more pronounced for 13-93 than that for S53P4. Further, an increase in the contact angle of both the SGs was observed postsilanization as per the grafting of the APTS. On the other hand, for PG, the contact angle reduced upon washing regardless of the pH of the buffer solution due to an increase in the exposure of OH groups on the phosphate glass surface when exposed to aqueous media, thus increasing its wettability. Upon silanization of PG, the contact angle increased to similar values to SG, in agreement with refs.^19,21,22^ As demonstrated in ref (22), an increase in contact angle upon silanization is representative of proper silane grafting on the surface of the glass disc.

### 3.3 Change in the Surface Charge due to Washing and Silanization

**Figure 4:**
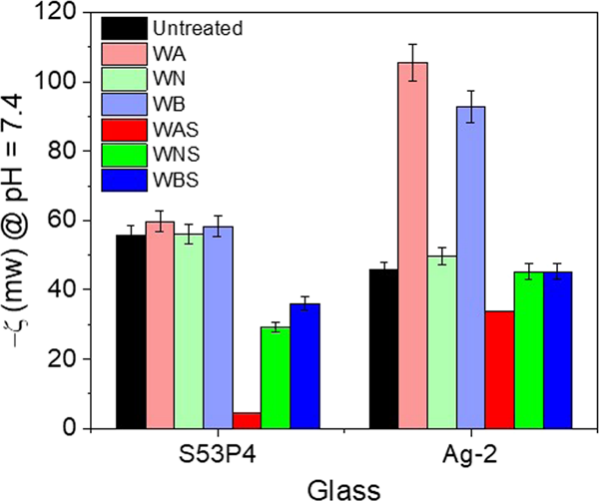
ζ-Potentials, taken at pH 7.4, of S53P4 and Ag-2, as examples, postwashing in various buffer solutions and postsilanization.

Figure 4 shows the changes in ζ-potential (at pH 7.4) on the surfaces of SG (S53P4) and PG (Ag-2) glasses, taken as representatives of their groups, as a function of washing in different buffers and postsilanization.

In the untreated condition, the ζ-potential was almost the same on the surface of both glasses. Upon washing, no change in the ζ-potential was evidenced on the surface of S53P4. However, postsilanization, an increase in ζ-potential was observed for all of the conditions, WA, WN, and WB, with a maximum for the WAS condition. For PG, the ζ-potential remained unchanged for the WN samples, whereas it decreased significantly for the WA and WB conditions. Additionally, a similar increase in the ζ-potential was evidenced postsilanization, which then remained constant irrespective of the pH of the buffer solution used for washing. A decrease in the ζ-potential indicates a more negatively charged surface, whereas an increase is due to a less negatively charged surface. In the case of SG (S53P4), the lack of change in ζ-potential indicates that the surface, as untreated, is most likely already saturated in OH^–^ groups. In the case of the silanized samples, the increase in ζ-potential is related to the presence of protonated amine groups from the APTS. In fact, APTES-modified surfaces are reported as positively charged at the physiological pH.^49−51^ The greater change in ζ-potential observed for the WAS SG may indicate that the presence of an HCA layer (Figure 1) leads to better APTS adsorption on the glass surface. Contrastingly, a sharp decrease in the ζ-potential of WA and WB Ag-2 samples was observed, which might be correlated to an increased OH– concentration on the material surface or depletion of the very top surface of the glass in cation, leading to negatively charged units on the glass surface.^22^ The increase in ζ-potential postsilanization is also an indication of the presence of protonated amines on the glass surface. The ζ-potential of the silanized PG is significantly higher than that of the corresponding SG due to the lower APTS grafting ability of PGs.^22^

### 3.4 Confocal Microscopy to Evidence Protein Adsorption

**Figure 5:**
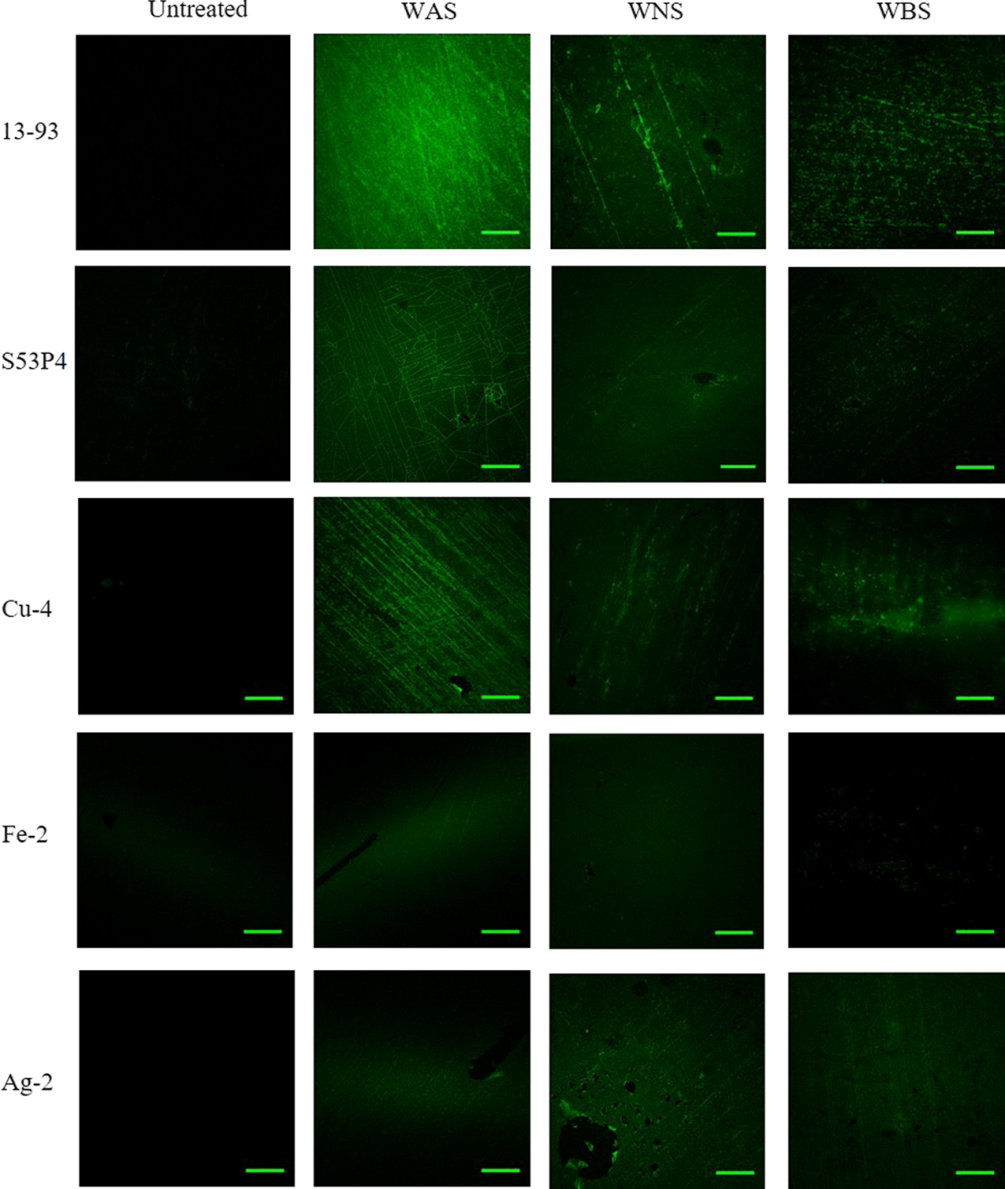
Confocal microscopy images of the glasses surface washed with various buffer solutions and silanized. The samples were further placed in contact with fluorescently labeled albumin. The scale bar represents 100 μm.

Model proteins, fluorescently labeled human fibronectin and BSA, were deposited on the surface of the untreated, washed, and washed + silanized glass discs. The adsorption of albumin was evidenced by confocal fluorescence microscopy (Figure S4 for the washed and Figure 5 for the washed + silanized samples).

The green fluorescence in the images can be assigned to the fluorescently labeled albumin proteins, as no autofluorescence was observed on the treated or untreated glass surfaces. Among all of the glasses in the untreated condition, only S53P4 depicted visible green fluorescence, whereas the surfaces of all of the other glasses appeared dark. On the other hand, all of the treated surfaces (washed and washed + silanized) exhibited protein adsorption as indicated by the green fluorescence. WAS 13-93 exhibit the maximum degree of green fluorescence. In addition, the WBS and WNS surfaces depict clusters of proteins on their surface, as opposed to the uniform fluorescence observed across the WAS samples. This might be related to the loosening of the BSA structure at pH between 7 and 9.^27^ For the PG, an improvement in protein adsorption was evidenced postwashing and washing+silanization. However, the degree of protein adsorption on the differently treated surfaces appears similar visually. Results were rather expected, as incubation in an aqueous, rather dilute protein, solution was expected to cause some surface degradation; 13-93 is a well-known silicate bioactive glass^52−54^ with a dissolution rate slower than S53P4^32^ and PG in this study. Therefore, the more stable surface of 13-93 provided a better substrate for protein adsorption as compared to other glasses.

**Figure 6:**
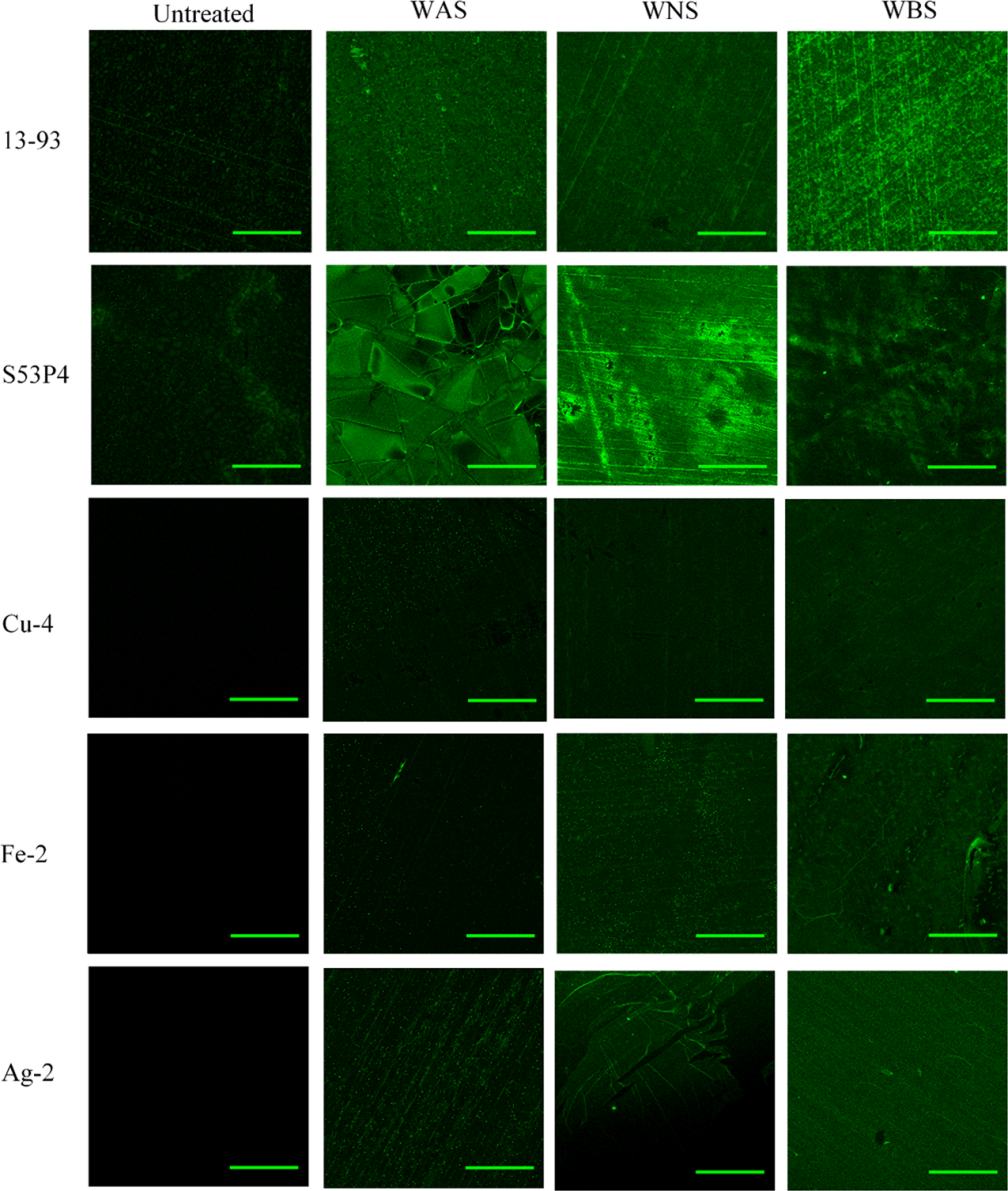
Confocal microscopy images of the glasses surface washed with various buffer solutions and silanized. The samples were further placed in contact with fluorescently labeled fibronectin. The scale bar represents 100 μm.

Figure 6 presents the confocal microscopy image of the glass discs’ surfaces treated (washed + silanized) with fluorescently labeled fibronectin as a function of surface treatment. Figure S5 presents the confocal microscopy images taken on washed glasses with fluorescently labeled fibronectin, for comparison. Similar to Figure 5, both the untreated SG depicted green fluorescence. Furthermore, the highest fluorescence could be observed in the WBS condition for 13-93 and the WNS condition for S53P4. Noticeably, WAS S53P4 exhibited a surface with unusual topological features, which could be assigned to the precipitation of the HCA layer. As for the PG, no fluorescence was evidenced in the untreated condition; however, all of the treated conditions depicted some degree of fluorescence, indicating that APTS promotes protein adsorption.

Here, it is important to note that different microscopes were used to image the fibronectin-coated glasses and BSA-coated glasses. Therefore, the results are not strictly comparable between the two protein groups.

### 3.5 Quantitative Analysis of the Confocal Images

**Figure 7:**
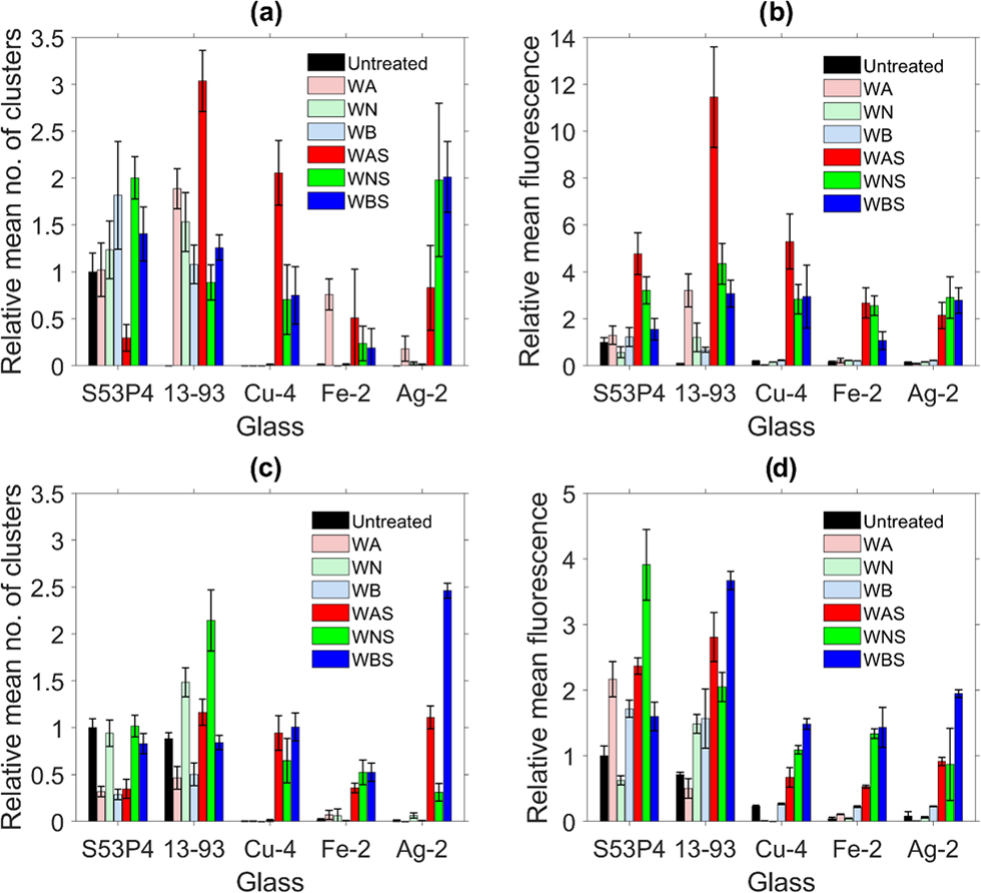
Albumin: (a) relative mean number of clusters and (b) relative mean total fluorescence of untreated, washed, and silanized SG and PG discs. Fibronectin: (c) relative mean number of clusters and (d) relative mean total fluorescence of untreated, washed, and silanized SG and PG discs. All values were presented relative to those for untreated S53P4. Vertical error bars correspond to the standard deviations, estimated as described in Section S3.

Quantitative analysis was performed to objectively compare and understand protein adsorption across the treatments. The relative total fluorescence and the relative number of clusters (as described in Section 2) were obtained from the confocal microscopy images. Figure 7a,b depicts the relative number of clusters (RNCs) and the relative total fluorescence (RTF) on the surface of the glass discs treated with BSA, respectively.

All of the values are presented relative to those for the untreated S53P4 sample. In the untreated condition, S53P4 exhibited the highest RNC among all of the samples, while the highest RNC overall was presented by the WAS 13-93 sample. For S53P4, the maximum RNC was observed for the WNS samples, whereas WAS samples showed the highest RNC in the case of 13-93. In the case of the PG, Cu-4, Fe-2, and Ag-2 presented the highest RNC on the surface of the WAS, WA, and WNS, respectively. S53P4 showed the highest RTF within the untreated samples (Figure 7b). Overall, from Figure 7, the RTF is maximum for all sample, except Ag-2, washed in acidic condition and silanized (WAS). In the case of Ag-2, washing in acidic buffer followed by silanization (WAS) yield a slightly lower RTF than when the surface is silanized postwashing in neutral or even basic conditions. Among the SG, the RTF increased in the WA condition, whereas among the PG, the RTF remained constant across all of the untreated and the washed samples but increased upon silanization. Noticeably, all of the PG depicted nearly zero RNC and RTF among the untreated samples, compared to the SG. Overall, Fe-2 presented the lowest RNC even in the washed and washed + silanized samples. A clear enhancement in the RNC and RTF for all of the SG was evidenced in the washed + silanized condition (Figure 7a,b). This is an indication of their inherent protein adsorption capacity relative to the PG, as even in the untreated condition, they show the ability to form clusters as well as adsorb higher amounts of proteins overall on their surface. The untreated PGs depict nearly zero clusters and protein adsorption on their surface. However, in the washed + silanized condition, PGs depict much stronger improvement in terms of RNC and RTF than the SG. Therefore, the ability of the PG to form clusters and adsorb proteins improved from nearly zero for the untreated condition to almost similar to that of the commercial SG postsilanization. Also, for RNC, no clear preference was observed for a particular treatment. On the other hand, a clear improvement in the RTF (a better indicator of the ability to adsorb higher density of proteins) was observed in the WAS samples, barring Ag-2. For Ag-2, the highest RTF was evidenced for the WBS and WNS samples.

The RNC and RTF were also obtained from the images of fibronectin-coated samples (Figure 7c,d). Among the untreated samples, S53P4 exhibited the highest RNC and RTF. Across all of the conditions, 13-93 and Ag-2 possessed the maximum RNC for SG and PG, respectively. Additionally, among the SG, the RNC improved for the WN samples and further increased for WNS 13-93. For the PG, the RNC remained nearly zero in the untreated and across all of the wash conditions and improved only after silanization. For Cu-4 and Ag-2, the maximum RNC was depicted by the WBS samples, while for Fe-2, it was the WNS samples. Across all of the glasses, Fe-2 exhibited the lowest RNC, even in the washed and washed+silanized conditions. In Figure 7d, overall, the SGs present a higher RNC than the PGs. In the case of the SG, the RTF increased very little upon washing, while the increase was more dramatic after silanization. On the other hand, for the PG also, nearly no increase in the RTF was observed postwashing, while the RTF increased dramatically postsilanization. Overall, the WBS samples presented the maximum RTF among all of the glasses, except for S53P4 that was the WNS samples. This is also corroborated from the FTIR spectra of the S53P4, where a stronger ability to precipitate HCA was found when compared to 13-93. This led to the precipitation of a much thicker layer of HCA on the surface of S53P4 as compared to that on 13-93. Since the ability of a glass to support cell growth and proliferation is assessed by its ability to precipitate an HCA layer, the thicker HCA layer may have resulted in higher protein adsorption on WNS S53P4 as compared to WNS 13-93. In addition, the confocal microscopy image of the WAS S53P4 sample depicted the precipitation of a highly crystalline layer, which is also in line with the observations from the FTIR spectra. It is worth noting that this HCA layer in the WAS S53P4 sample appeared broken on and away from the surface, while that on the WNS S53P4 was more uniform.

From the ζ-potential results, the higher ζ-potential on S53P4 and Ag-2 was evidenced in the WAS condition. It is clear from the image analysis that albumin was more efficiently adsorbed onto these samples, indicating that this protein is more likely to bind on less negatively charged surfaces. On the other hand, WBS samples, for both SG and PG, appear to be the more negatively charged of the silanized samples and result in greater fibronectin adsorption.

In summary, we showed that protein adsorption on the surface of phosphate glasses can be improved by customizing the surface charge and grafting silane on their surface. This result is of relevance in view of the need for further understanding the key role of protein adsorption on cell attachment and proliferation on a biomaterial. For each protein, a specific wash condition was found to lead to favorable surface properties for protein adsorption. Protein adsorption improved further with silanization. This may pave way for a new generation of surface-treated phosphate glasses, with improved protein adsorption.

## 4 Conclusions

In this work, traditional silicate (S53P4 and 13-93) and phosphate glasses within the metaphosphate composition (Ag-2, Cu-2, Fe-2) were surface-treated in view of improving protein adsorption. Treatments involved washing the glass surface with buffers with pH 5.0, 7.4, and 9.0 as such a range should not induce significant protein denaturation. As expected, washing with basic and neutral buffer did not significantly affect the surface chemistry of the silicate glasses. However, washing with acidic buffer led to early precipitation of a reactive layer, established to be hydroxyapatite. Such a phenomenon agrees with a faster dissolution of silicate glasses at acidic pH. In the case of the phosphate glasses, the congruent dissolution leads to unchanged surface chemistry, regardless of the buffer used. While the washing does not seem to impact drastically the contact angle nor the ζ-potential of silicate bioactive glasses, a decrease in contact angle and an increase in ζ-potential could be seen for the phosphate glasses.

Successively, a silanization step using APTS was conducted to graft amine groups on the glass surface. The surface chemistry (FTIR) of the glass substrates was not affected by the silanization process. However significant changes in contact angle and ζ-potential were reported. Indeed, regardless of the glass composition, APTS grafting led to an increase in the hydrophobicity and a decrease in the net surface charge due to the presence of protonated amine on the material surface.

Albumin and fibronectin were adsorbed on the glass surface. When the glasses were untreated, proteins could only be adsorbed on the surface of the SG. A simple washing step in buffers of various pH levels improved mildly the protein adsorption. However, silanization was effective in promoting protein adsorption on the surface of the phosphate glasses, with adsorption similar to the one on the surface of the silicate bioactive glasses. It is interesting to point out that albumin adsorption was more effective on materials washed in acidic buffer and further silanized, while fibronectin was more efficiently adsorbed on the surface of glasses washed with basic buffer solution and further silanized. The reasons for such prevalence of various proteins to be grafted on materials treated at different pH levels are not yet well understood and will be investigated in the future. However, one point that should be taken into consideration is surface chemistry. Indeed, the glass 13-93 treated may have promoted more protein adsorption due to its more stable chemistry when compared to S53P4 and PBGs. However, in the case of S53P4 washed in the acidic buffer, the surface chemistry completely changed from an amorphous silicate to HA, which will have strong implications toward protein adsorption. Furthermore, it is assumed that washing in a basic solution increases the OH– group, thus leading to more bonding sites for APTES and explaining the improvement of fibronectin adsorption. In the case of albumin, it is well accepted that the basic pH may lead to loosening of the protein structure, thus leading to clustering. In acid, no such changes in conformation are reported, leading to a more uniform coverage of the glass surface.

## Supporting Information Available

Figure S1, Representative image of a drop on the surface of the S53P4-WBS; Figure S2, example of the results of protein clusters’ segmentation for the 13-93 WBS glass surface; Section S3, variability of the number of clusters and total image fluorescence between subregions as a function of the number of subregions; Figure S4, confocal microscopy images of the glass surface washed with various buffer solution; and Figure S5, confocal microscopy images of the glasses surface washed with various buffer solutions (PDF)
