# Supplementary material for: Surface Modification of Bioresorbable Phosphate Glasses for Controlled Protein Adsorption
Source: ACS Biomater Sci Eng. 2021 Aug 12;7(9):4483–93. doi: 10.1021/acsbiomaterials.1c00735 (PMC8441970; doi:10.1021/acsbiomaterials.1c00735)
Supplement: Supplementary file 1 — ab1c00735_si_001.pdf [file ab1c00735_si_001.pdf]

Supplementary files

Surface modification of bioresorbable phosphate glasses for controlled protein adsorption

Ngoc Bao Hyunh[1], Cristina Santos Dias Palma[2], Rolle Rahikainen[3], Ayush Mishra[1], Latifeh Azizi[3], Enrica Verne[4], Sara Ferraris[4], Vesa Pekka Hytönen[3,5], Andre Sanches Ribeiro[2], Jonathan Massera[1*]

[1]*Laboratory of Biomaterials and Tissue Engineering, Faculty of Medicine and Health Technology, Tampere University, Korkeakoulunkatu 3, 33720 Tampere, Finland*

[2]*Laboratory of Biosystem Dynamics, Faculty of Medicine and Health Technology, Tampere University, Tampere, Finland*

[3]*Laboratory of Protein Dynamics, Faculty of Medicine and Health Technology, Tampere University, Arvo Ylpön katu 34, 33520 Tampere, Finland*

[4]Laboratory of Biomaterials, Department *of Applied Science and Technology, Politecnico di Torino, 24 Corso Duca Degli Abruzzi, 10129 Torino, Italy*

[5]*Fimlab Laboratories, Biokatu 4, 33520 Tampere, Finland*

* Corresponding author: Jonathan Massera, jonathan.massera@tuni.fi

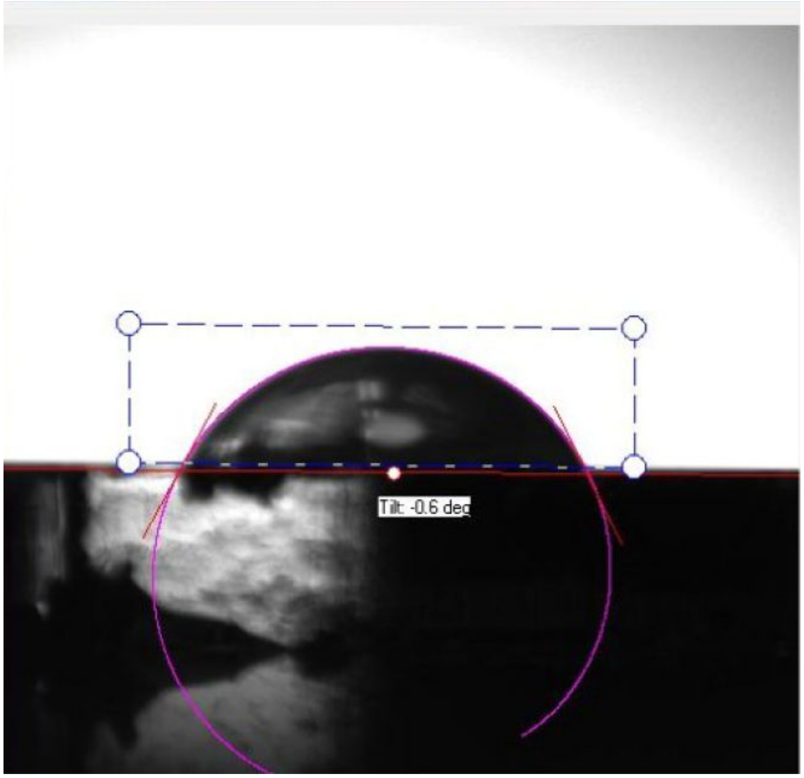

Figure S1: representative image of a drop on the surface of the S53P4-WBS

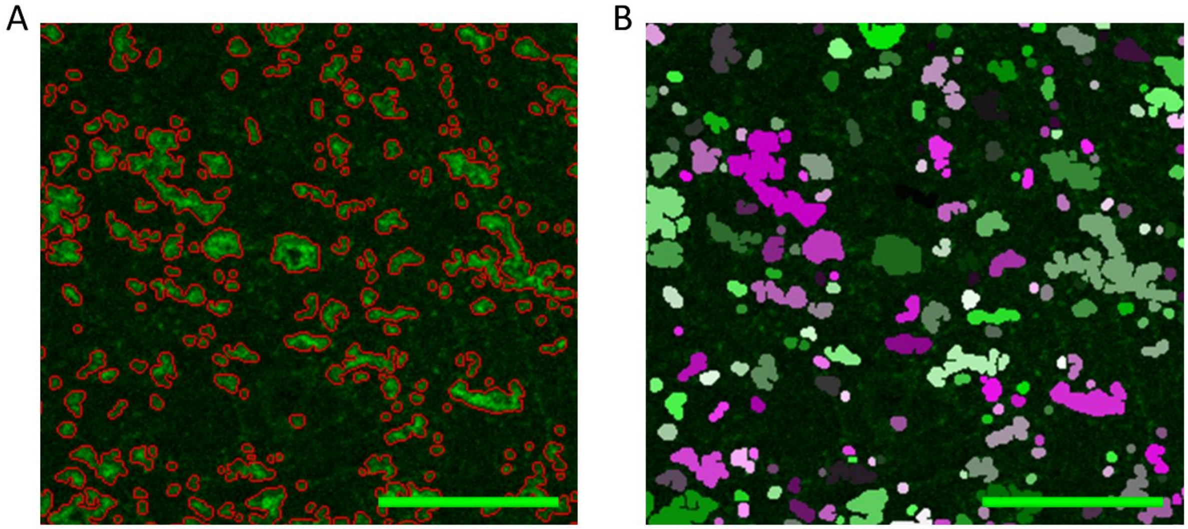

Figure S2: Example of the results of protein clusters' segmentation for the glass surface 13-93, WBS. (A) Results of automatic cluster segmentation. Red lines represent the borders of the (green) clusters identified. (B) The area (mask) of each cluster identified in (A) was colored, in order to visually determine the quality of the segmentation. Scale bar represents 50 µm.

Supplementary Section S3:

We expect variability in the numbers of protein clusters and total fluorescence between regions of an image, due to intrinsic and extrinsic noise sources. Intrinsic sources generate variability between sub-regions of an image. These sources are (but not limited to) noise in protein binding, spreading of total fluorescent protein per region, and segmentation of the clusters.

Meanwhile, the only expected significant extrinsic sources of noise (i.e. causing variability between different images, but not between sub-regions) is variability between surfaces and the total amount of fluorescent protein placed on each surface. We found these extrinsic sources to be negligible, when compared to the variability produced by the intrinsic sources.

Based on this, we next describe how we quantify the standard deviation of the number of clusters ($STD_{clusters}$) and of the total image fluorescence ($STD_{intensity}$) from 3 example images. First, to decide the size of the sub-regions of the image, we selected sub-regions by randomly selecting a pixel in the image. That pixel is then used as the upper left corner of a square with *L x L* dimensions (in pixels), for increasing *L*. These sub-regions were obtained by bootstrapping with replacement the full image, with the number of selected sub-regions being equal to *N*, where *N* is the integer of the ratio between the area of the full image and the area of the sub-region (*L x L*). We note that higher and lesser amounts of sampling than *N* result in sub- and over-sampling of the full image, respectively.

In Figures S3D and S3E, we show the estimations of the variability of the number of clusters and total protein fluorescence using this methodology, for increasing *N*. We find that the results for the three example images only become discernible when having multiple sub-regions (N > 20), showing that accounting for extrinsic noise alone would not suffice to distinguish values of $STD_{clusters}$ and $STD_{intensity}$ of the three example images (in agreement with the expectations for low extrinsic noise).

Meanwhile, as we split the image into an increasing number of sub-regions, it emerges a significant difference between them that becomes quasi-stable at *N* ~ 25. Finally, for *N* > 45, the 3 lines eventually become again indistinguishable, as the sub-regions become smaller than the average cluster size. As such, we opted for obtaining N = 25, equally-sized sub-regions per image, for estimating the variability in number of clusters and in total image fluorescence. The resulting sub-image have a size that suffices for multiple clusters to fit in, across all conditions.

We obtained similar results if the sub-regions are instead obtained by equally dividing the full image (insets Figures S3D and S3E). However, since this method does not allow equally dividing the whole image by 25 sub-regions, we opted for the method above. Results are shown in Figure 7 (main manuscript).

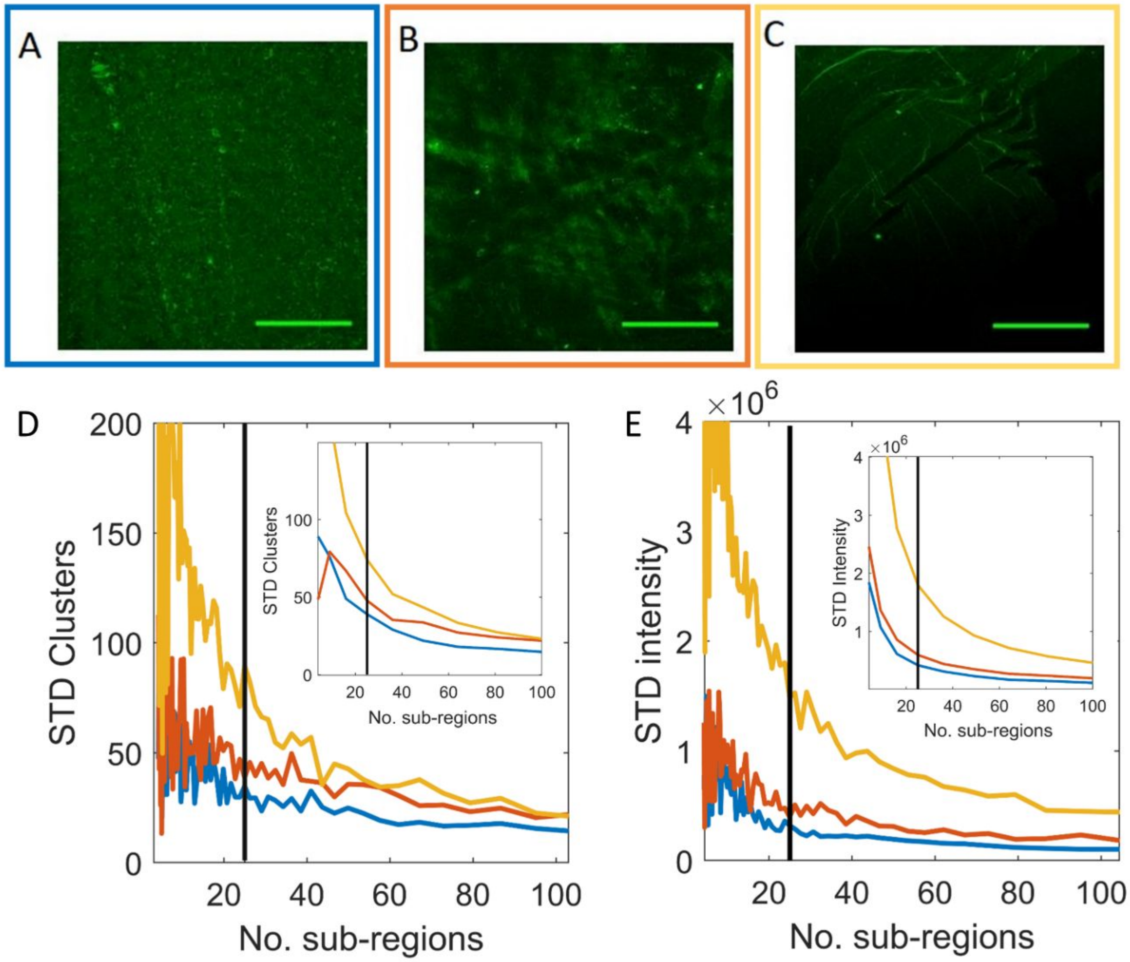

Figure S3: Variability of the number of clusters and total image fluorescence between sub-regions as a function of the number of sub-regions as estimated from **(A-C)** representative images of the data. Scale bars represents 100 µm. **(D)** Standard deviation of the number of clusters as a function of the number of sub-regions per image, when sub-regions are obtained by bootstrapping of the full image. **(D inset)** Standard deviation of the number of clusters as a function of the number of sub-regions per image, when sub-regions are obtained by equally diving the full image. **(E)** Standard deviation of the total fluorescence of the sub-regions as a function of the number of sub-regions per image, when sub-regions are obtained by bootstrapping of the full image. **(E inset)** Standard deviation of the total fluorescence of the sub-regions as a function of the number of sub-regions per image, when sub-regions are obtained by equally diving the full image. In figures D and E, the blue, orange, and yellow lines are obtained from the analysis of images A, B and C, respectively. The black vertical line corresponds to the number of sub-regions (25) selected to perform the variability analysis in figure 7 of the main manuscript.

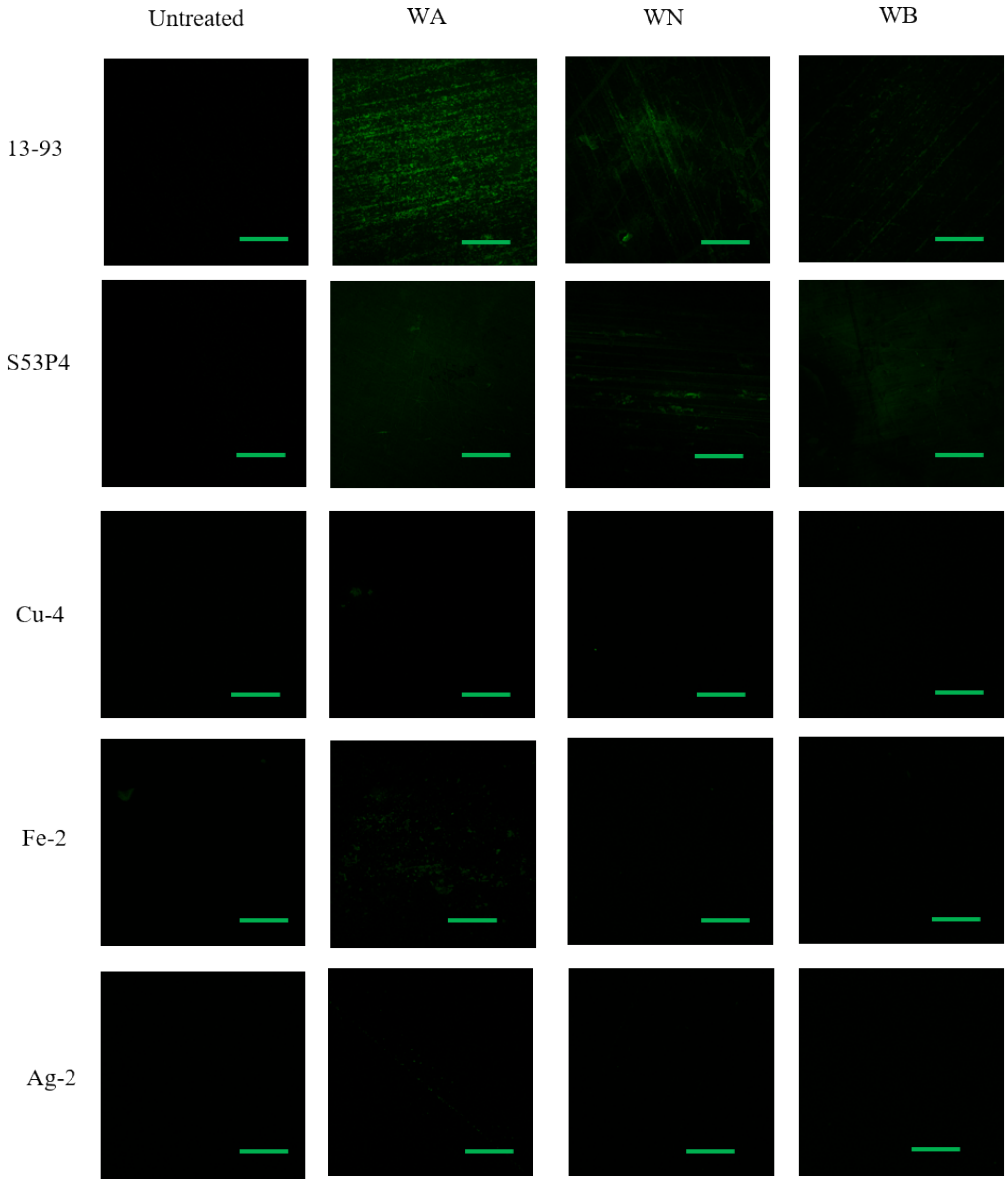

Figure-S4 Confocal microscopy images of the glasses surface washed with various buffer solutions. The samples were further placed in contact with fluorescently labelled albumin. Scale bar represents 100 µm.

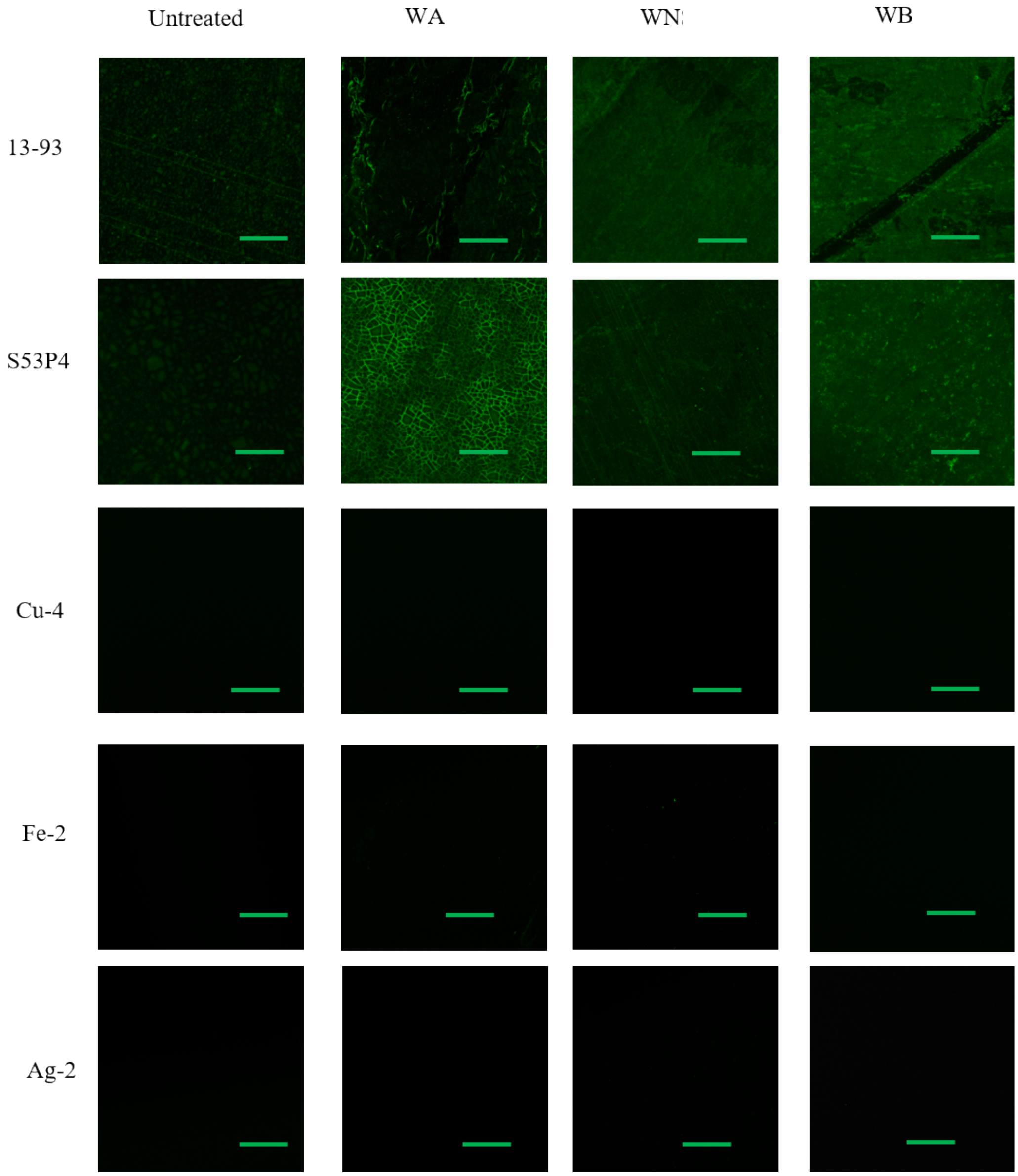

Figure-S5 Confocal microscopy images of the glasses surface washed with various buffer solutions. The samples were further placed in contact with fluorescently labelled Fibronectin. Scale bar represents 100 μm.
